# Temporal evolution of clinical event risk after primary-prevention ICD implantation: Chagas vs. non-Chagas dilated cardiomyopathy

**DOI:** 10.3389/fcvm.2026.1950508

**Published:** 2026-09-16

**Authors:** Juan Pablo Guzmán, Pedro Diaz Uberti, Camila Rosello, Maribel Capobianco, Valeria Piazza, Fernando Di Tommaso, Javier Zubiri, Francisco Toscano

**Affiliations:** 1 Arrhythmia and Cardiac Pacing Section, Department of Cardiology, Hospital Churruca-Visca, Buenos Aires, Argentina; 2 Electrophysiology Service, Hospital Rivadavia, Buenos Aires, Argentina; 3 Department of Cardiology, Hospital Rivadavia, Buenos Aires, Argentina

**Keywords:** Chagas cardiomyopathy, competing risks, implantable cardioverter-defibrillator, restricted mean survival time, risk stratification, sudden cardiac death, ventricular arrhythmia

## Abstract

Chagas cardiomyopathy evolves through progressive myocardial injury that may alter the temporal distribution of arrhythmic risk compared with other dilated substrates. We analysed a two-centre registry of consecutive primary-prevention implantable cardioverter-defibrillator (ICD) recipients in Buenos Aires (2008–2025), excluding cardiomyopathies in which ICD indication is not based on ventricular dysfunction. The final cohort comprised 194 patients: 50 with Chagas cardiomyopathy (26%), 83 with ischaemic (43%) and 61 with non-ischaemic dilated cardiomyopathy (31%). Median LVEF was 30% in both groups. The primary endpoint was a composite of appropriate ICD therapy, adjudicated on stored electrograms, or all-cause death. Pre-planned analyses included multivariable Cox regression, period-specific models split at 5 years, competing-risk regression and restricted mean survival time (RMST). The endpoint occurred in 109 patients. Cumulative incidence was lower in Chagas patients at 1 year (2.1% vs. 12.2%) and 5 years (19.5% vs. 42.4%), and Chagas patients accumulated 682 additional event-free days at 10-year RMST (95% CI: 258–1047; *p* = 0.001). Chagas cardiomyopathy was associated with lower composite risk (hazard ratio 0.58; 95% CI: 0.37–0.91; *p* = 0.018), an association confined to the first 5 years (0.35; 0.17–0.75; *p* = 0.007) and no longer detectable thereafter (0.85; 0.47–1.54; *p* = 0.587), with 28 and 56 patients at risk at 5 years and 11 and 20 at 10 years. The wide confidence interval and small population at risk in the late period mean that absence of a detectable difference should not be read as evidence of equivalent risk. These findings are hypothesis-generating and require confirmation in larger cohorts.

## 1 Introduction

Chagas cardiomyopathy, the leading non-ischaemic cause of sudden cardiac death in Latin America (1, 2), develops through progressive patchy fibrosis and conduction disease that set it apart from ischaemic and idiopathic dilated cardiomyopathy (3). The arrhythmogenic substrate is not fixed at a single point in time. It expands as inflammation and fibrosis accumulate over years to decades, producing an anatomical basis for reentry that grows in parallel with disease duration. ICD guidelines, by contrast, are anchored to left ventricular ejection fraction (LVEF) thresholds (4, 5), which capture static ventricular function at the moment of assessment but are blind to the temporal kinetics of substrate evolution. The Rassi score, the most validated risk tool for Chagas cardiomyopathy (6), predicts mortality but was not designed to describe how arrhythmic hazard changes over the years following ICD implantation.

Chagas disease is no longer geographically contained. It affects an estimated 6–7 million people worldwide, and although prevalence is falling in endemic countries, it is rising in non-endemic regions, driven mainly by migration, which demands new strategies for screening, early recognition, and access to care (7). Migration has established a 3.5% pooled seroprevalence among Latin American migrants in Europe (8) and an estimated 55,000 infected individuals in Spain (9). Electrophysiologists outside endemic regions increasingly manage these patients without a clear evidence base for long-term device follow-up, and the problem is compounded in the public health systems that care for most Chagas patients, where continuity of device surveillance competes with constrained resources. Meta-analyses of ICD outcomes in Chagas cardiomyopathy have reported conflicting results, with appropriate therapy rates ranging from 27% to 55% depending on the comparator group and the duration of follow-up (10, 11). This heterogeneity is itself a clue: if arrhythmic risk in Chagas were constant over time, follow-up duration would not so strongly determine the reported event rate.

The remaining question concerns the temporal kinetics of this risk. Does progressive fibrosis shift the arrhythmic hazard curve rightward, generating a delayed pattern of risk accumulation rather than the fixed, early-peaking risk characteristic of ischaemic substrate? The clinical stakes are direct. If arrhythmic risk in Chagas peaks late, a study or a surveillance protocol that ends at three to five years will miss the highest-risk window and will underestimate the importance of maintaining long-term device surveillance. We used a two-centre long-term registry of primary-prevention ICD recipients, with follow-up extending to 17 years, to characterise the temporal dynamics of arrhythmic risk in Chagas cardiomyopathy.

## 2 Materials and methods

### 2.1 Study population and design

This was a retrospective analysis of a prospectively maintained registry of consecutive ICD recipients at two public electrophysiology referral centres in Buenos Aires, Argentina (Hospital Churruca-Visca and Hospital Rivadavia), enrolled between January 2008 and December 2025. Both centres serve the same national public health system and apply a homogeneous implantation and follow-up protocol, which reduces the between-centre variability in access and device management that can confound multicentre registries. Inclusion criteria were the presence of an implantable cardioverter-defibrillator implanted for primary prevention of sudden cardiac death and at least one documented follow-up visit. Exclusion criteria were implantation for secondary prevention; cardiomyopathies and inherited arrhythmia syndromes in which ICD indication is not based on ventricular dysfunction and in which ejection fraction is characteristically preserved; and absence of follow-up data. Of 225 patients assessed for eligibility, 31 met an exclusion criterion, leaving a final cohort of 194 patients (Figure 1). This is a registry of device carriers rather than a study of implantation indication: eligibility was defined by the presence of an implanted device, and implantation decisions had been made prospectively under the guideline recommendations in force at the time, across an enrolment period spanning seventeen years. The study was approved by the institutional review boards of both centres, and the requirement for informed consent was waived owing to the retrospective design. No prospective sample-size calculation was performed; the cohort comprises all consecutive eligible patients over the enrolment period.

**Figure 1:**
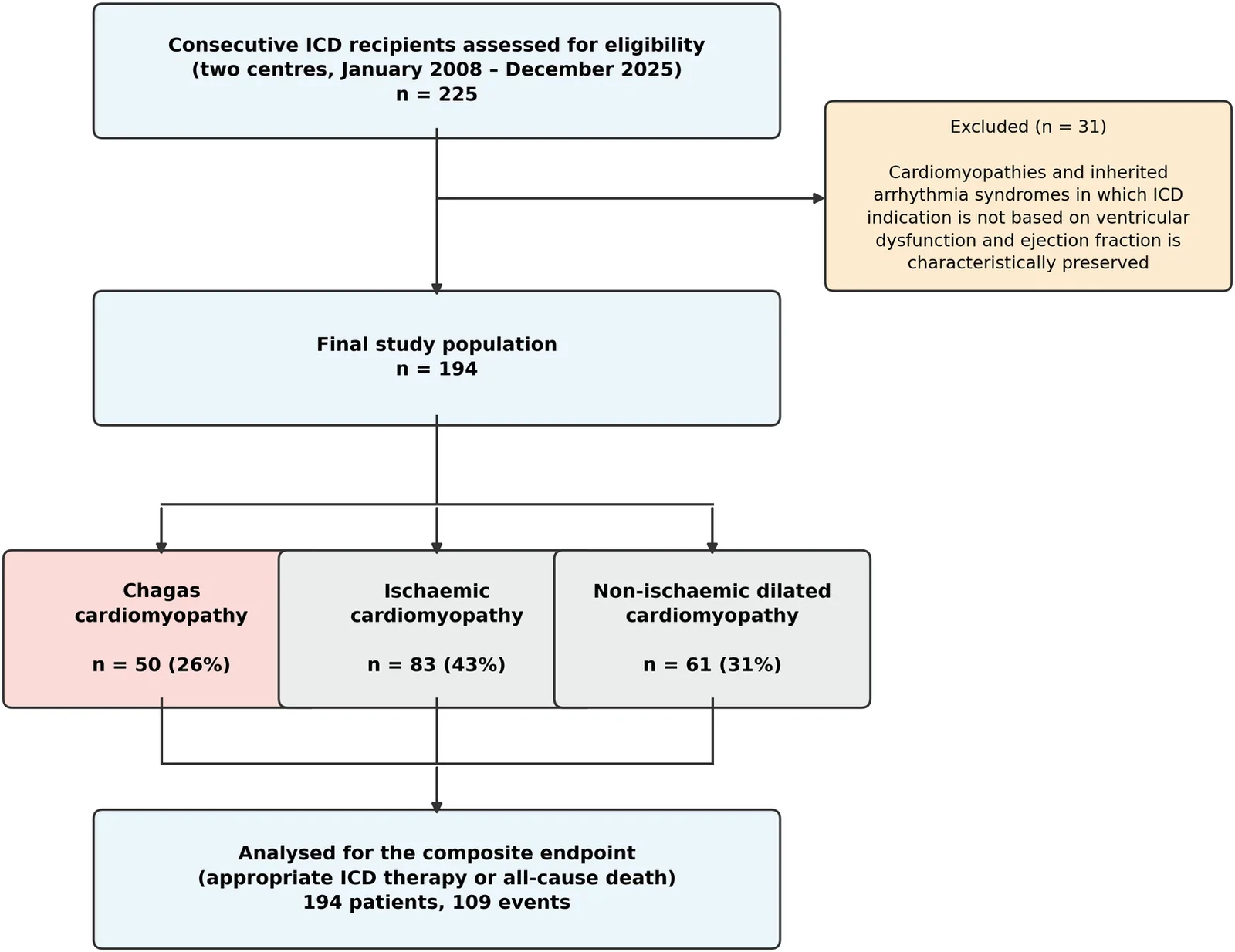
Study flow diagram.

### 2.2 Chagas diagnosis and aetiological classification

Chagas seropositivity was confirmed by two orthogonal serological assays, combining ELISA with indirect haemagglutination or indirect immunofluorescence, in accordance with Argentine national guidelines (12), which require agreement between two distinct methods for a positive diagnosis. Chagas cardiomyopathy was assigned as the primary aetiology on the basis of serology together with a compatible clinical and imaging phenotype. The compatible clinical and imaging phenotype was defined as segmental or global left ventricular dysfunction together with conduction abnormalities characteristic of the disease. Coronary disease of a severity sufficient to explain the ventricular dysfunction was an exclusion for the Chagas classification; a minority of Chagas patients had mild concomitant coronary disease that was not considered the cause of their cardiomyopathy. Ejection fraction is reported from the echocardiographic study closest to implantation available in the registry. The non-Chagas group comprised ischaemic cardiomyopathy (*n* = 83) and non-ischaemic dilated cardiomyopathy (*n* = 61).

### 2.3 Endpoint and event adjudication

The primary endpoint was a composite of first appropriate ICD therapy or all-cause death, whichever occurred first. Appropriate ICD therapy was defined as antitachycardia pacing or shock delivered for documented ventricular tachycardia or ventricular fibrillation. Every device-delivered therapy was adjudicated independently by two electrophysiologists through review of the stored device electrograms, and only therapies judged appropriate by both reviewers were counted as events; inappropriate therapies, for atrial arrhythmias, oversensing, or lead noise, were not counted. This dual, electrogram-based adjudication reduces the misclassification of arrhythmic events that can arise when device-generated reports are accepted without electrogram review. The composite endpoint was prespecified because a patient who dies before receiving appropriate therapy cannot contribute to an arrhythmia-only endpoint, which would systematically undercount arrhythmic burden and bias a cause-specific comparison between groups with differing mortality.

### 2.4 Statistical analysis

Continuous variables are presented as mean ± SD and, for ejection fraction, also as median and interquartile range, since the distribution is not symmetrical. Categorical variables are presented as proportions. Between-group comparisons used the Student *t*-test and the Fisher exact test. Kaplan–Meier curves were compared with the log-rank test. Restricted mean survival time (RMST) at 10 years was pre-specified as the primary estimand, because it does not assume proportional hazards and expresses the treatment contrast as a difference in event-free time, which is clinically interpretable (13). Competing-risk cumulative incidence functions were estimated by the Aalen-Johansen method for each endpoint component separately (14).

Multivariable Cox regression adjusted for LVEF (per SD), non-sustained ventricular tachycardia (NSVT), and amiodarone use. With 109 events and four covariates, the events-per-variable ratio was 27.2, well above the conventional threshold for stable estimation. The proportional-hazards assumption was assessed using scaled Schoenfeld residuals, reported under both the default Kaplan–Meier and a logarithmic time transform, since the two can differ and reporting only one would not be reproducible. Because the study hypothesis concerned a change in hazard over time, two temporal analyses were pre-planned on biological grounds rather than being triggered by a statistical test. First, period-specific Cox models split follow-up at 5 years, with the late period conditioned on survival to 5 years. The 5-year threshold was defined *a priori*, before inspection of the survival curves, for two reasons: it approximates the median follow-up of the published Chagas ICD series whose divergent findings motivated this analysis, and it corresponds approximately to the service life of a defibrillator generator, which is the interval over which decisions about continued surveillance are made in practice. Sensitivity analyses using cut-points at 3 and 7 years are reported in Supplementary Table S1. Second, a time-varying Cox model including a Chagas × log(time) interaction was fitted using counting-process notation. Because the estimate from this model proved sensitive to the choice of split points and time transform, it is reported as a sensitivity analysis rather than as a confirmatory test, with the full range of specifications given in Supplementary Material. Covariates were selected *a priori* on clinical grounds and constrained by the number of events. Ejection fraction and NSVT were chosen because primary-prevention indication and arrhythmic risk stratification rest principally on them. Amiodarone was included for a reason specific to this population: in Latin American practice, patients with moderate- to high-risk Chagas cardiomyopathy characteristically receive amiodarone either alongside a defibrillator or as sole therapy, frequently for economic reasons, and amiodarone also modifies the probability of the endpoint itself by raising the threshold at which the device delivers therapy. An extended model additionally including QRS duration, sex and age is reported in Supplementary Table S2. Fine-Gray subdistribution regression, with death treated as a competing event (15), estimated the association between Chagas cardiomyopathy and appropriate ICD therapy. Analyses were performed in Python 3.12 (lifelines 0.29) and R 4.3 (survival, cmprsk). The de-identified dataset and analysis scripts are available from the corresponding author upon reasonable request.

## 3 Results

### 3.1 Baseline characteristics

Of 194 patients, 50 (26%) had Chagas cardiomyopathy, 83 (43%) ischaemic cardiomyopathy and 61 (31%) non-ischaemic dilated cardiomyopathy. Chagas patients were more often female (40% vs. 14%; *p* < 0.001) and had wider QRS (127.7 ± 27.2 vs. 116.8 ± 28.9 ms; *p* = 0.018). Ejection fraction did not differ between groups (32.3 ± 14.6% vs. 34.7 ± 14.0%; *p* = 0.325), with a median of 30% in both (IQR: 23–40 and 25–41). NSVT and amiodarone use were similar. Follow-up was longer in the Chagas group (6.8 ± 5.7 vs. 5.0 ± 4.5 years; *p* = 0.020), reflecting the earlier position of these patients within the enrolment period (Table 1).

**Table 1:** Baseline characteristics.

Variable	Chagas (*n* = 50)	Non-Chagas (*n* = 144)	*p* value
Age at implant (years)	62.5 ± 12.1	61.4 ± 12.5	0.590
Female sex	20 (40%)	20 (14%)	<0.001
LVEF (%)	32.3 ± 14.6; median 30 (IQR: 23–40)	34.7 ± 14.0; median 30 (IQR: 25–41)	0.325
QRS duration (ms)	127.7 ± 27.2	116.8 ± 28.9	0.018
NSVT at implant	25 (50%)	68 (47%)	0.745
Amiodarone use	22 (44%)	80 (56%)	0.189
Follow-up, years (mean ± SD)	6.8 ± 5.7	5.0 ± 4.5	0.020
Follow-up, median (IQR)	5.8 (3.1–9.4)	3.4 (1.2–8.1)	
Composite endpoint	25 (50.0%)	84 (58.3%)	0.325
Appropriate ICD therapy	17 (34.0%)	53 (36.8%)	0.864
All-cause death	15 (30.0%)	53 (36.8%)	0.492
Aetiology			
Ischaemic cardiomyopathy	—	83 (58%)	
Non-ischaemic dilated cardiomyopathy	—	61 (42%)	

Continuous variables are mean ± SD; categorical variables are proportions. *p* values are from the Student *t*-test or Fisher exact test. LVEF, left ventricular ejection fraction; NSVT, non-sustained ventricular tachycardia.

### 3.2 Event rates and temporal trajectories

The composite endpoint occurred in 109 patients (56.2%): 25 of 50 Chagas patients (50.0%) and 84 of 144 non-Chagas patients (58.3%). The timing of events differed markedly (Figure 2A). Cumulative incidence in Chagas vs. non-Chagas patients was 2.1% vs. 12.2% at 1 year, 19.5% vs. 42.4% at 5 years, and 51.6% vs. 71.5% at 10 years. Over the 10-year horizon, Chagas patients accumulated 682 additional event-free days (95% CI: 258–1047; *p* = 0.001), an estimate that does not depend on the proportional-hazards assumption. Because precision declines as the population at risk falls, RMST was also estimated at earlier horizons: +129 days at 3 years (95% CI: 52–199), +276 days at 5 years (95% CI: 125–424) and +445 days at 7 years (95% CI: 194–680). The difference was significant at every horizon, and the widening confidence interval reflects the diminishing number of patients contributing information over time (Supplementary Table S3). Numbers at risk, together with cumulative events and censored patients at each interval, are given in Figure 2 and Supplementary Table S4.

**Figure 2:**
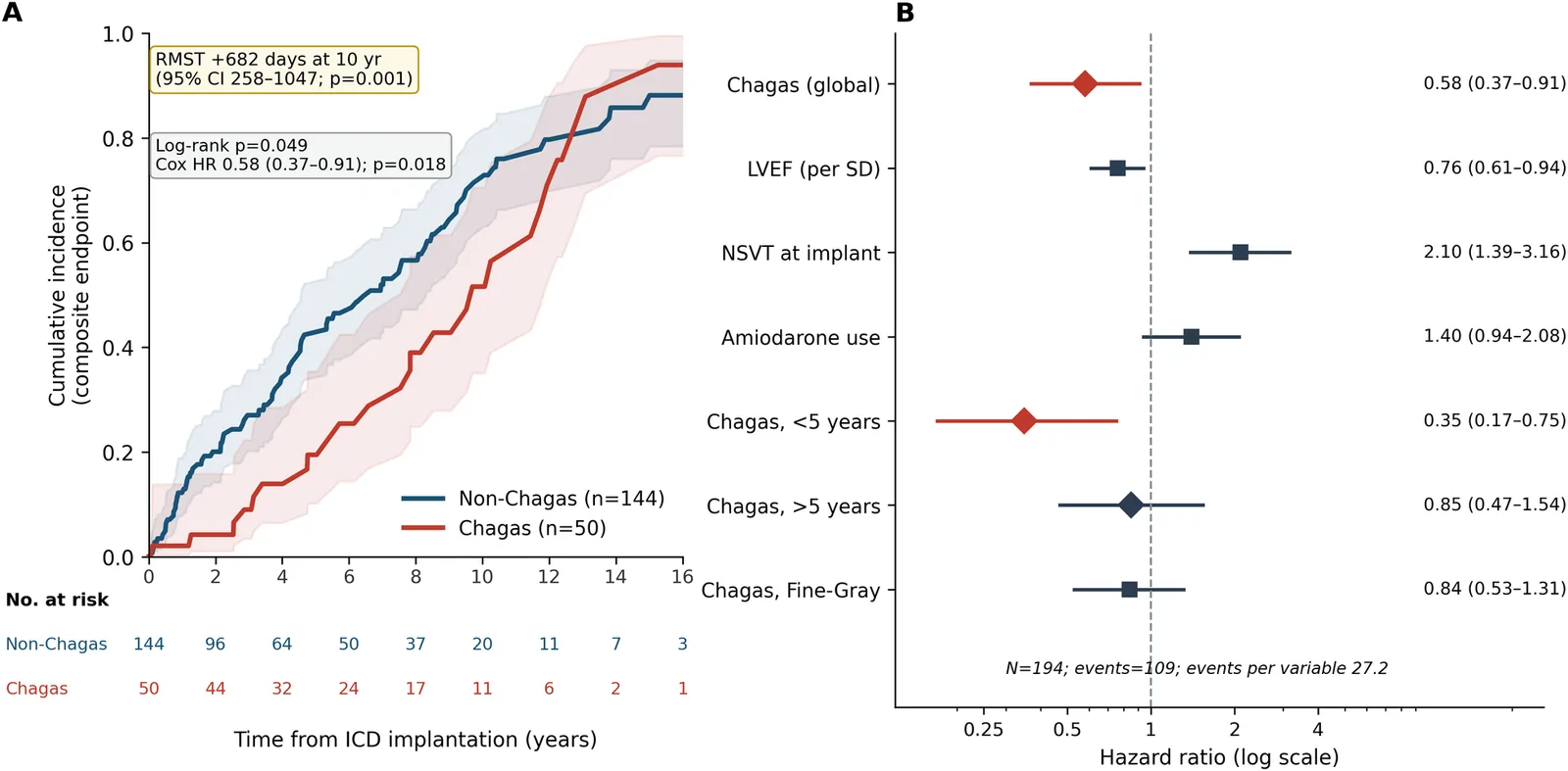
Time-varying pattern of the composite endpoint. **(A)** Cumulative incidence (1 minus Kaplan–Meier survival) of the composite endpoint in Chagas (red, *n* = 50) and non-Chagas (blue, *n* = 144) patients, with the 10-year RMST difference (+682 days; 95% CI: 258–1047; *p* = 0.001). **(B)** Multivariable Cox forest plot (*N* = 194; events = 109; events per variable 27.2), showing the global Chagas hazard ratio, covariates, period-specific Chagas hazard ratios, and the Fine-Gray subdistribution hazard ratio with death as a competing event.

### 3.3 Multivariable and temporal models

In multivariable Cox regression, Chagas cardiomyopathy was independently associated with lower composite risk (HR: 0.58; 95% CI: 0.37–0.91; *p* = 0.018). Ejection fraction (HR: 0.76 per SD; 95% CI: 0.61–0.94; *p* = 0.014) and NSVT (HR: 2.10; 95% CI: 1.39–3.16; *p* < 0.001) were the other independent predictors. Amiodarone was associated with numerically higher risk without reaching significance (HR: 1.40; 95% CI: 0.94–2.08; *p* = 0.097). Schoenfeld residuals indicated a violation of the proportional-hazards assumption for Chagas aetiology (*p* = 0.015 using the default Kaplan–Meier time transform; *p* = 0.12 using a logarithmic transform; global *p* = 0.009), consistent with a hazard ratio that changes over time and providing formal support for the pre-planned period-specific analyses (Table 2, Figure 2B). The association was confined to the first 5 years: HR: 0.35 (95% CI: 0.17–0.75; *p* = 0.007; 58 events). Beyond 5 years no difference was detectable: HR: 0.85 (95% CI: 0.47–1.54; *p* = 0.587; 51 events) (Figure 3), with 28 Chagas and 56 non-Chagas patients at risk at the 5-year landmark. The confidence interval for the late period is wide and compatible with a range of effects, so the absence of a detectable difference in this period should not be interpreted as evidence that the risks have become equivalent. A time-varying model including a Chagas × log(time) interaction did not reach significance (*p* = 0.213); because this estimate is sensitive to the choice of split points, the full range of specifications is reported in Supplementary Table S5. The landmark analysis (Supplementary Figure S1) plots this trajectory continuously: the adjusted Chagas hazard ratio was below 0.5 in the early years and approached 1.0 after year 8. A sensitivity analysis restricted to patients with an ejection fraction of 35% or below (*n* = 129) preserved all principal estimates (Supplementary Table S6).

**Figure 3:**
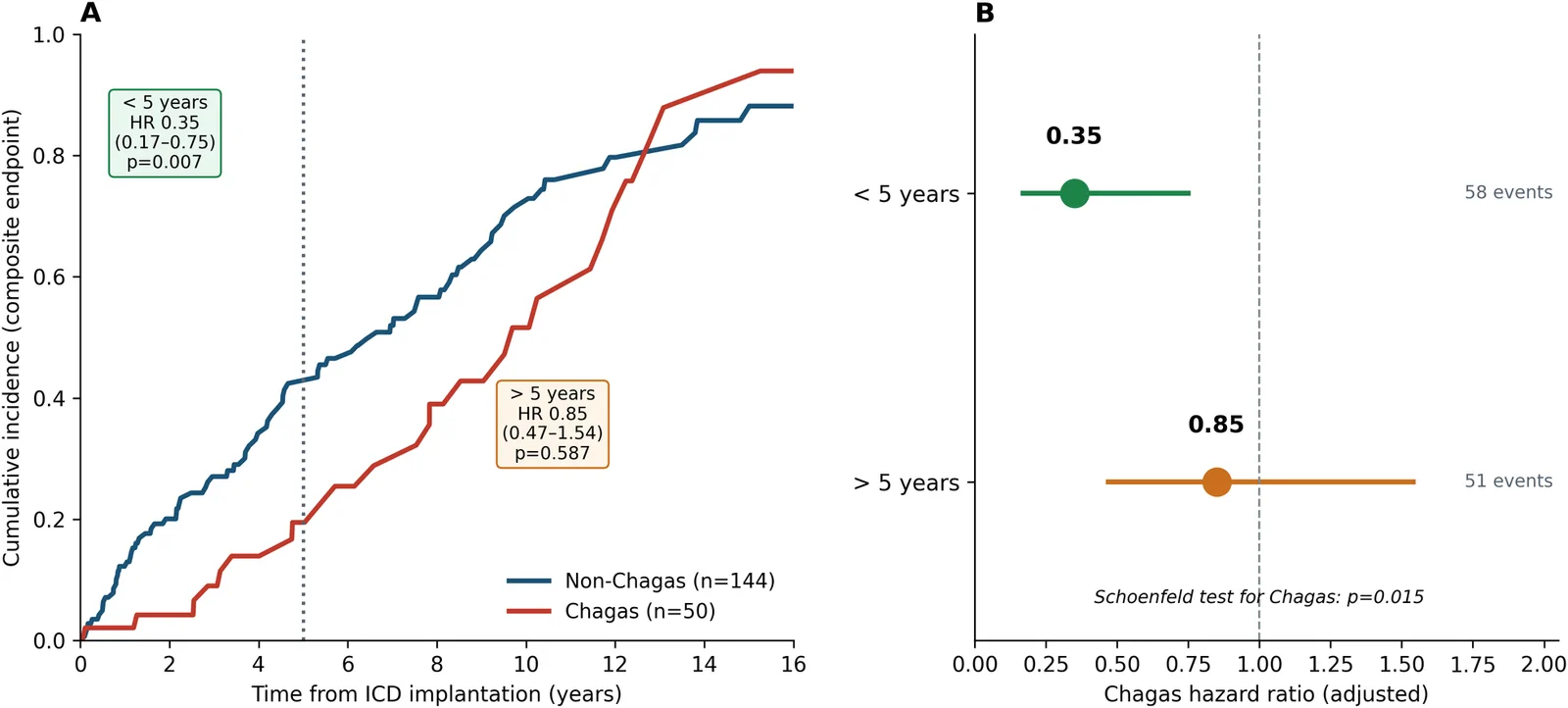
Period-specific hazard. **(A)** Kaplan–Meier curves with period-specific adjusted hazard ratios (green, <5 years; orange, >5 years). **(B)** Chagas hazard ratio by period. The proportional-hazards (Schoenfeld) test for Chagas aetiology was significant (*p* = 0.015), consistent with a time-varying effect.

**Table 2:** Multivariable regression results.

Variable/Model	HR	95% CI	*p* value
Global model (*N* = 194, events = 109, EPV = 27.2)			
Chagas cardiomyopathy	0.58	0.37–0.91	0.018
LVEF (per SD)	0.76	0.61–0.94	0.014
NSVT at implant	2.10	1.39–3.16	<0.001
Amiodarone use	1.40	0.94–2.08	0.097
Period-specific analysis (Chagas vs Non-Chagas)			
<5 years (*N* = 194, events = 58)	0.35	0.17–0.75	0.007
>5 years (*N* = 84, events = 51)	0.85	0.47–1.54	0.587
Time-varying and competing-risk models			
Fine-Gray (ICD therapy; death competing)	0.84	0.53–1.31	0.430

All Cox models were adjusted for LVEF (per SD), NSVT at implant, and amiodarone use. The period-specific late model was conditioned on survival beyond 5 years. HR, hazard ratio; CI, confidence interval; EPV, events per variable.

### 3.4 Competing-risk analysis

Cause-specific cumulative incidence functions (Supplementary Figure S2) showed appropriate ICD therapy in 34.0% of Chagas and 36.8% of non-Chagas patients, and all-cause death in 30.0% and 36.8%. Neither component differed in absolute rate, consistent with the composite reflecting a temporal redistribution of events rather than a difference in total burden. The near-identical final rates for each component, combined with the divergent timing of the composite, indicate that the two groups reach a similar endpoint by different routes and on different schedules. Non-Chagas patients reached 50% event accrual by year 4.2, and Chagas patients by year 7.8 (Supplementary Figure S3), a difference of more than three years in the median timing of events. Fine-Gray regression treating death as a competing event attenuated the Chagas association for appropriate ICD therapy alone (subdistribution HR: 0.84; 95% CI: 0.53–1.31; *p* = 0.430). The arrhythmia-specific association was therefore directionally consistent with the composite but did not reach significance, so the temporal pattern is established for the composite endpoint and remains uncertain for arrhythmic events in isolation.

### 3.5 Analysis by aetiology

Taking Chagas cardiomyopathy as reference, the adjusted hazard for the composite endpoint was 1.76 (95% CI: 1.08–2.86; *p* = 0.022) in ischaemic and 1.68 (95% CI: 0.99–2.86; *p* = 0.055) in non-ischaemic dilated cardiomyopathy. In pairwise comparisons the Chagas hazard ratio was 0.55 (95% CI: 0.33–0.89; *p* = 0.015) against ischaemic and 0.54 (95% CI: 0.31–0.94; *p* = 0.029) against non-ischaemic cardiomyopathy. The similarity of these estimates indicates that the early separation of the curves is not attributable to the ischaemic component of the comparator (Supplementary Table S7).

## 4 Discussion

The principal finding concerns the timing of events rather than their total number. Chagas cardiomyopathy was associated with roughly half the composite event rate of non-Chagas patients during the first 5 years, whereas beyond 5 years no between-group difference was detectable, with considerably less precise estimates. At 10 years, the net result was 682 additional event-free days. A study limited to three to five years would capture a marked early separation between groups, whereas longer follow-up would show that this difference is no longer statistically detectable. The latter should not be interpreted as evidence that the risks have become equivalent. This time dependence may help explain part of the heterogeneity in a literature that has reported divergent Chagas ICD outcomes, since prior series sampled different segments of the follow-up.

These findings sit within a literature in which the temporal distribution of ICD benefit has long been recognised as non-uniform. In MADIT-II the survival advantage of the defibrillator was not apparent in the early months and emerged progressively during follow-up (16), and in SCD-HeFT the survival curves separated only after approximately one year (17). Neither trial enrolled patients with Chagas cardiomyopathy, and neither addressed whether the arrhythmic hazard itself follows a different trajectory according to aetiology. CHAGASICS (18), the only randomised trial in this population, compared amiodarone against a defibrillator rather than optimal medical therapy with and without a device, a design that has been questioned because in regional practice these treatments coexist rather than compete (19). Our cohort reflects that reality, with amiodarone widely used alongside device therapy, and this is the reason amiodarone was retained in the adjusted model.

One biologically plausible interpretation concerns the substrate itself. The arrhythmogenic substrate in Chagas is built progressively. Chronic myocarditis, microvascular injury, and autonomic denervation drive patchy fibrosis that matures into the slow-conduction channels required for sustained reentry, and this process continues over years to decades after diagnosis (1–3). The ischaemic substrate behaves differently (4): a fixed peri-infarction scar is largely established early, so arrhythmic risk peaks in the first years and then declines. This divergence in substrate kinetics is compatible with the pattern we observed, a lower early hazard in Chagas that rises as the fibrotic process advances. We had anticipated that the ischaemic component of the comparator, with its early-peaking arrhythmic risk, would amplify the early contrast. The aetiology-specific analysis does not support this: the Chagas hazard ratio was almost identical against ischaemic and against non-ischaemic cardiomyopathy (0.55 and 0.54). The early separation may therefore relate to the Chagas substrate itself rather than to the composition of the comparator group. Gondim et al., in a 207-patient cohort conducted over an 18-year period, reported higher rates of appropriate ICD therapies and mortality in Chagas than in non-ischaemic cardiomyopathy, with a median follow-up of approximately five years (20). Differences in comparator composition, prevention indication, and follow-up distribution may contribute to the contrast with our findings. Vieira de Melo et al. reported worse one-year prognosis in Chagas after secondary-prevention implantation (21), a different indication and a different time horizon from the primary-prevention question addressed here.

Two structural explanations must also be considered. Patients who reach ICD implantation are survivors, and those with the highest early arrhythmic burden may die or be identified before enrolment, which would lower the apparent early event rate in the Chagas group. The timing of implantation relative to disease onset also matters, because a patient implanted early in the fibrotic process will show lower early event rates than one implanted after decades of progression, and this information is not captured in our registry. Competing risks operate throughout follow-up: the CHAGASICS trial showed that ICDs halved sudden death but did not reduce total mortality in Chagas (18), so part of the arrhythmic benefit is absorbed by non-arrhythmic death. More broadly, observational evidence in ICD recipients has shown that associations with appropriate device therapy may change when death is explicitly modelled as a competing event (22). The attenuation of the Chagas association in the Fine-Gray model (subdistribution HR: 0.84) is consistent with the influence of death as a competing event.

The clinical implication follows from the shape of the hazard, not from its average. Within the observed follow-up, LVEF and NSVT were the strongest predictors of events, although Chagas aetiology was also independently associated with lower composite risk (HR: 0.58; *p* = 0.018). LVEF-guided criteria for who should receive an ICD remain valid. The potential clinical relevance of these findings relates to the interpretation of an event-free early course. Our data suggest that early event-free survival in Chagas cardiomyopathy should not automatically be assumed to identify a persistently low-risk long-term trajectory. Whether this temporal pattern should influence the intensity or duration of device surveillance requires prospective evaluation. We emphasise that different surveillance strategies were not compared in this registry, and that this is a potential clinical implication requiring confirmation in larger independent cohorts rather than a management recommendation established by the present data. A patient who has been event-free for five or six years has not necessarily entered a low-risk phase, because our data do not demonstrate that the early between-group difference persists during later follow-up. Our data address the timing of events and not decisions about generator replacement, which were not evaluated here and require dedicated study. VT catheter ablation is an emerging adjunct in this population, and recent data suggest that worse long-term outcomes after VT ablation in Chagas cardiomyopathy may be driven predominantly by mortality rather than by greater VT recurrence (23), which is consistent with the competing-risk picture described here. Functional substrate ablation may nevertheless reduce VT recurrence, while early post-ablation recurrence identifies patients at increased risk of death (24).

It is worth stating separately what these data show and what they do not. What the analysis demonstrates is that, among primary-prevention ICD recipients with dilated cardiomyopathy, Chagas aetiology was associated with a substantially lower composite event rate during the first five years, amounting to 682 additional event-free days over a ten-year horizon, and that this difference was no longer detectable thereafter. What remains a hypothesis is that progressive evolution of the arrhythmogenic substrate underlies this pattern: it is biologically plausible and consistent with what is known of the disease, but it is not established by these data. What remains speculative is any causal attribution to myocardial fibrosis, which was neither imaged nor quantified longitudinally in this registry; the longer QRS duration observed in the Chagas group is consistent with more extensive structural and electrical involvement, but conduction delay is not a surrogate for fibrosis.

### 4.1 Limitations

This study has several limitations. It is observational, and the Chagas subgroup comprises 50 patients, so the precision of period-specific estimates is limited, particularly beyond five years, where 28 Chagas and 56 non-Chagas patients remained at risk and the confidence intervals are correspondingly wide. Follow-up was longer in the Chagas group, reflecting their earlier position within the enrolment period, and differential follow-up duration bears on the interpretation of period-specific estimates. Ejection fraction was taken from the echocardiographic study closest to implantation available in the registry. NYHA functional class, ventricular aneurysm and detailed imaging findings were not recorded systematically across the full enrolment period, and beta-blockers, sotalol and heart-failure therapies could not be included in the adjusted model for the same reason; residual confounding by these factors cannot be excluded. Amiodarone use is subject to confounding by indication, since it is prescribed to patients with a greater arrhythmic burden, and its estimate should not be read causally. Cause-specific mortality was not adjudicated. ICD programming criteria evolved across the 2008–2025 enrolment window. Only 8 patients were followed beyond 15 years, so estimates in the far tail of the curves rest on few observations. The retrospective observational design precludes causal inference: the temporal pattern described here is an association observed within a registry, not a demonstrated effect of aetiology on arrhythmic risk over time. The findings should be regarded as hypothesis-generating and require confirmation in larger, independent prospective cohorts.

## 5 Conclusion

In primary-prevention ICD recipients with dilated cardiomyopathy, Chagas aetiology was associated with a lower early composite-event risk. This difference was no longer detectable beyond five years, although the late-period estimates remain imprecise because of the small number of patients at risk during that interval, and the absence of a detectable difference should not be read as evidence of equivalent risk. Arrhythmia-specific analyses were directionally consistent but did not reach significance. Favourable early outcomes should not be assumed to indicate a persistently low-risk trajectory; whether this should influence long-term device surveillance warrants evaluation in larger prospective cohorts.

## Data Availability

The raw data supporting the conclusions of this article will be made available by the authors, without undue reservation.
