# Supplementary material for: Temporal evolution of clinical event risk after primary-prevention ICD implantation: Chagas vs. non-Chagas dilated cardiomyopathy
Source: Front Cardiovasc Med. 2026 Sep 16;13:1950508. doi: 10.3389/fcvm.2026.1950508 (PMC13623618; doi:10.3389/fcvm.2026.1950508)
Supplement: Supplementary file 1 [file Datasheet1.docx]

**Supplementary Material**

*Temporal Evolution of Clinical Event Risk After Primary-Prevention ICD Implantation: Chagas Versus Non-Chagas Dilated Cardiomyopathy*

Guzmán JP, Diaz Uberti P, Rosello C, Capobianco M, Piazza V, Di Tommaso F, Zubiri J, Toscano F

**1 Supplementary Tables**

**Supplementary Table 1. Sensitivity of the period-specific models to the choice of cut-point**

All models adjusted for LVEF (per SD), NSVT and amiodarone use. The late period is conditioned on survival beyond the cut-point.

| **Cut-point** | **Early period, Chagas HR** | **Late period, Chagas HR** |
| --- | --- | --- |
| 3 years | 0.29 (0.10–0.81); p=0.019 | 0.70 (0.42–1.18); p=0.184 |
| 5 years (primary) | 0.35 (0.17–0.75); p=0.007 | 0.85 (0.47–1.54); p=0.587 |
| 7 years | 0.40 (0.21–0.76); p=0.005 | 0.91 (0.46–1.78); p=0.781 |

*The pattern of a lower early hazard followed by attenuation is preserved across cut-points, indicating that the finding does not depend on the choice of five years.*

**Supplementary Table 2. Extended multivariable Cox model**

Primary model with QRS duration, sex and age added. Adding these covariates reduces the events-per-variable ratio from 27.2 to 15.6.

| **Variable** | **HR** | **95% CI** | **p value** |
| --- | --- | --- | --- |
| Chagas cardiomyopathy | 0.55 | 0.34–0.89 | 0.015 |
| LVEF (per SD) | 0.76 | 0.60–0.96 | 0.021 |
| NSVT at implant | 2.02 | 1.33–3.07 | 0.001 |
| Amiodarone use | 1.41 | 0.94–2.11 | 0.095 |
| QRS duration (per SD) | 0.99 | 0.80–1.22 | 0.899 |
| Male sex | 0.95 | 0.55–1.62 | 0.839 |
| Age at implant (per SD) | 1.14 | 0.93–1.40 | 0.196 |

*QRS duration, sex and age did not contribute materially, and the Chagas estimate was essentially unchanged. The parsimonious model was therefore retained as primary.*

**Supplementary Table 3. Restricted mean survival time across horizons**

Difference in event-free time, Chagas minus non-Chagas. Confidence intervals from 2000 bootstrap replicates.

| **Horizon** | **Difference (days)** | **95% CI** | **p value** | **Patients at risk** |
| --- | --- | --- | --- | --- |
| 3 years | +129 | 52 to 199 | 0.004 | 139 |
| 5 years | +276 | 125 to 424 | <0.001 | 84 |
| 7 years | +445 | 194 to 680 | <0.001 | 64 |
| 10 years | +682 | 258 to 1047 | 0.001 | 31 |

*The difference is significant at every horizon and the direction is consistent. The confidence interval widens as the number of patients contributing information declines.*

**Supplementary Table 4. Patients at risk, cumulative events and censored patients**

| **Year** | **Chagas at risk** | **Chagas events** | **Chagas censored** | **Non-Chagas at risk** | **Non-Chagas events** | **Non-Chagas censored** |
| --- | --- | --- | --- | --- | --- | --- |
| 0 | 50 | 0 | 0 | 144 | 0 | 0 |
| 1 | 47 | 1 | 2 | 118 | 17 | 9 |
| 2 | 44 | 2 | 4 | 96 | 27 | 21 |
| 3 | 38 | 4 | 8 | 80 | 35 | 29 |
| 4 | 32 | 6 | 12 | 64 | 42 | 38 |
| 5 | 28 | 8 | 14 | 56 | 50 | 38 |
| 6 | 24 | 10 | 16 | 50 | 54 | 40 |
| 7 | 21 | 11 | 18 | 43 | 59 | 42 |
| 8 | 17 | 14 | 19 | 37 | 63 | 44 |
| 10 | 11 | 17 | 22 | 20 | 74 | 50 |
| 12 | 6 | 21 | 23 | 11 | 79 | 54 |
| 15 | 2 | 24 | 24 | 6 | 82 | 56 |
| 17 | 1 | 25 | 24 | 1 | 83 | 60 |

*Only 8 patients were followed beyond 15 years and 2 beyond 17 years. Estimates in the far tail of the survival curves therefore rest on few observations.*

**Supplementary Table 5. Sensitivity of the time-varying interaction**

Chagas × log(time) interaction under four specifications of the counting-process model, all fitted to the same cohort.

| **Model construction** | **p value** | **Interpretation** |
| --- | --- | --- |
| Cuts 1, 2, 3, 5, 7, 10, 15 / log(interval midpoint) | 0.213 | not significant |
| Cuts 1, 2, 3, 5, 7, 10, 15 / log(interval end) | 0.520 | not significant |
| Cuts 1, 3, 5, 10 / log(interval midpoint) | 0.354 | not significant |
| Single cut at 5 years / log(interval end) | 0.009 | significant |

*Because the estimate depends on modelling choices that were not pre-specified, the interaction is reported as a sensitivity analysis and not as a confirmatory test. The temporal pattern rests on the period-specific models, on the proportional-hazards diagnostics, and on RMST, which are stable across specifications.*

**Supplementary Table 6. Analysis restricted to patients with LVEF 35% or below**

Sensitivity analysis restricted to patients meeting the conventional guideline threshold at the recorded assessment.

| **Result** | **Primary cohort (n=194)** | **LVEF ≤35% (n=129)** |
| --- | --- | --- |
| Chagas patients | 50 | 33 |
| Composite events | 109 | 71 |
| Log-rank p | 0.049 | 0.020 |
| Cox global, Chagas HR | 0.58 (0.37–0.91); p=0.018 | 0.43 (0.23–0.79); p=0.007 |
| Patients at risk at 10 years | 31 | 13 |

*All principal estimates are preserved and strengthened in the restricted analysis. It was not adopted as the primary analysis because it reduces the population at risk beyond five years to a degree that would not support the temporal question addressed by the study.*

**Supplementary Table 7. Aetiology-specific analyses**

Adjusted for LVEF (per SD), NSVT and amiodarone use. The three-group model takes Chagas cardiomyopathy as reference; pairwise models compare Chagas with one comparator aetiology at a time.

| **Model / Comparison** | **HR** | **95% CI** | **p value** |
| --- | --- | --- | --- |
| **Three-group model (reference: Chagas cardiomyopathy)** | | | |
| Ischaemic cardiomyopathy | 1.76 | 1.08–2.86 | 0.022 |
| Non-ischaemic dilated cardiomyopathy | 1.68 | 0.99–2.86 | 0.055 |
| **Pairwise comparisons (Chagas as index group)** | | | |
| Chagas vs ischaemic (n=133, events=75) | 0.55 | 0.33–0.89 | 0.015 |
| Chagas vs non-ischaemic (n=111, events=59) | 0.54 | 0.31–0.94 | 0.029 |

*The two pairwise estimates are almost identical, indicating that the early separation of the event curves is not attributable to the ischaemic component of the comparator group.*

**2 Supplementary Figures**

**Supplementary Figure 1.** Dynamic hazard ratio and proportional-hazards diagnostics. (A) Adjusted Chagas hazard ratio as a function of landmark time, with 95% confidence band. The estimate is furthest from unity in the early years and approaches unity thereafter. (B) Scaled Schoenfeld residuals for the Chagas covariate against log(time). The proportional-hazards test for Chagas aetiology suggested non-proportionality under the default Kaplan-Meier time transform (p=0.015), although this was not robust to a logarithmic transform (p=0.12); both are reported so that the analysis is reproducible.

**Supplementary Figure 2.** Competing-risk cumulative incidence functions (Aalen-Johansen). (A) Cumulative incidence of appropriate ICD therapy, treating death as a competing event. (B) Cumulative incidence of all-cause death, treating appropriate ICD therapy as a competing event. Crude observed proportions over the whole follow-up were 34.0% versus 36.8% for ICD therapy and 30.0% versus 36.8% for all-cause death in Chagas and non-Chagas patients respectively.

**Supplementary Figure 3.** Precision of the RMST estimate and event accrual. (A) Difference in restricted mean survival time at four horizons, with 95% bootstrap confidence intervals and the number of patients at risk at each horizon. (B) Cumulative percentage of all events accrued over time; dashed lines mark the time at which 50% of events had occurred in each group (non-Chagas year 4.2, Chagas year 7.8).

**3 Supplementary Methods**

**Missing data.** Missing values were confined to one or two observations per variable and were imputed with the group-specific median before modelling. No multiple imputation was performed given the negligible proportion of missing data.

**Period-specific models.** Follow-up was split at 5 years. The early model included all patients with events censored at the cut-point. The late model was restricted to patients remaining event-free and under observation beyond the cut-point, with time re-set to zero at that landmark.

**Time-varying models.** Counting-process notation was used, splitting each patient record at the cut-points listed in Supplementary Table 5, with an interaction term between Chagas status and the natural logarithm of the interval midpoint or interval end as specified.

**Restricted mean survival time.** Estimated from the Kaplan-Meier curve of each group up to the specified horizon. Confidence intervals and p values were obtained by resampling both groups independently with replacement, 2000 replicates, with a fixed random seed.
